# The 3Rs and Humane Experimental Technique: Implementing Change

**DOI:** 10.3390/ani9100754

**Published:** 2019-09-30

**Authors:** Robert C. Hubrecht, Elizabeth Carter

**Affiliations:** UFAW, the Old School, Brewhouse Hill, Wheathampstead, Hertfordshire AL4 8AN, UK; carter@ufaw.org.uk

**Keywords:** the 3Rs, the Three Rs, replacement, reduction, refinement, alternatives, housing and husbandry, laboratory animals

## Abstract

**Simple Summary:**

The 3Rs: Replacement, Reduction and Refinement, formulated by William Russell and Rex Burch, have become synonymous with the measures to improve the welfare of animals used in research and are now used as an ethical framework for improving laboratory animal welfare throughout the world. This introduction to a special issue on the 3Rs explains how a non-confrontational and scientific approach led to their development, and briefly summarizes their adoption around the world.

**Abstract:**

In 1959, the Universities Federation for Animal Welfare (UFAW) Scholars Russell & Burch published the Principles of Humane Experimental Technique in which they laid out the principles of the Three Rs. However, the Three Rs owed much to others. It was UFAW and, in particular, UFAW’s Founder and Director, Major Charles Hume who identified the problem that needed to be tackled, and who developed the non-confrontational approach that was needed to both formulate the questions that needed answers and to obtain the answers from the research community. Russell & Burch’s work was also guided by an expert scientific and technical committee chaired by the Nobel Prize winner Sir Peter Medawar. This essay describes the history of the Three Rs using publications by the protagonists and others as well as material from UFAW’s archives. It describes the background to the employment of Russell & Burch, the methodology of Russell & Burch’s approach and the impact of their work up to the present day—where the Three Rs are incorporated in legislation throughout the world.

## 1. Introduction

The use of animals for research purposes (drug testing, models of disease, safety testing, or for the advancement of knowledge) has long been a matter of controversy, with concerns raised about animals’ sentience, the, sometimes, very significant harms that might be caused to them and whether the justification for such harms is acceptable. Most researchers working in the biosciences field and who use live animals in research will have heard of the Three Rs principles and understand that implementing them is the accepted way to address at least some of these concerns. Some will be aware of the impact that the Three Rs principles have had on the welfare of animals used in research and that they are now internationally accepted as the ethical framework under which animal experimentation should be conducted. However, many may not know that the Three Rs were developed as much as 60 years ago and that, although animal research was deeply controversial, they came out of a uniquely collaborative approach between an academic animal welfare organisation and the scientific community.

The Three Rs, as originally proposed by the Universities Federation for Animal Welfare (UFAW) scholars Russell and Burch [1] (*The Principles*), were defined as:

Replacement: The substitution for conscious living higher animals of insentient material;

Reduction: Reduction in the number of animals used to obtain information of given amount and precision;

Refinement: Any decrease in the severity of inhumane procedures applied to those animals, which still have to be used.

Russell and Burch described them in this order reflecting the order in which they should be addressed: (1) that sentient animals should not be used if non-sentient alternatives are available; (2) if animals do have to be used, then the project’s design and analysis should be such that the minimum number of sentient animals are used compatible with achieving the objectives of the research; and (3) that appropriate measures should be taken to mitigate any pain suffering or distress that the animals might feel.

The Three Rs have remained much the same since their original publication, although there have been minor changes of emphasis and of definition. For example, Russell & Burch were much occupied with achieving reduction by reducing variation due to differences in the animals’ genotype, interactions with environmental variables such as temperature, and by dealing with disease, which was an important issue at that time. Today, the health status of animals used in research is usually far better defined and maintained (e.g., [2]) and environmental variables are better controlled, so more emphasis is usually placed on achieving Reduction through good and appropriate experimental design. Research techniques have also evolved since *The Principles*. For example, with the development of transgenic research in the 1980s welfare concerns were raised, that were largely based on unpredictable effects and the numbers of animals involved [3,4]. However, various publications have considered the welfare assessment of genetically modified animals and ways to refine techniques involved in their production e.g., [5,6,7,8,9] and the welfare issues raised by genetic modification are not so different from those that arise in other areas of research [10,11]. As Russell & Burch wrote in *The Principles* “As new fields of biology open in the future, it may become a matter of routine to apply the lessons of the past and turn as soon as possible to the techniques of replacement”.

Another area where the emphasis has changed relates to animal care. Refinement is often implemented by either choosing less severe research protocols, or by ameliorating their effect by, for example, the use of appropriate anaesthesia or analgesia. However, the husbandry and handling of animals used in research or for breeding stock can also cause significant distress if it fails to meet their needs. It is clear that this was understood by Russell & Burch, as they wrote that “we cannot even in principle separate husbandry from the conduct of the experiment itself”. Nonetheless, since the publication of The Principles, Refinement has sometimes been defined in ways that have either omitted or appeared to omit husbandry [12] and this has led to a suggested redefinition of Refinement that: (1) clearly includes both breeding and research animals; (2) emphasises the importance of husbandry as well as changes to the design that impact on welfare and; (3) incorporates the concept it is important to consider how to positively impact well-being as well as minimise adverse effects on the animals [12].

## 2. The Genesis of the Three Rs

As in so many other areas of scientific endeavour, the Three Rs concept was not entirely novel. Richmond describes how some of the concepts, or parts thereof, were already being promoted many years earlier [13,14]. Marshal Hall, in 1831, in an introduction to his volume on the circulation of the blood [15] and also in 1847 [16] describes five principles for the prosecution of physiology, which embodies much of the Three Rs, and the need for necessary ethical justification:

1. We should never have recourse to experiment in cases which observation can afford us the information required.
2. No experiment should be performed without a distinct and definite object and without the persuasion, after the maturest consideration, that the object will be attained and produce a real and uncomplicated result.
3. We should not needlessly repeat experiments.
4. That it should be instituted with the least possible infliction of suffering.
5. Every physiological experiment should be performed under such circumstances as will secure due observation and attestation of its results, and so obviate, as much as possible, the necessity for its repetition.

The concept that animal use should be avoided (later encompassed by the Principles of Reduction and Replacement) also appears in an 1839 editorial in the London Medical Gazette, which advised that live animals should not be used:

… till it is sufficiently clear that the fact pursued neither is, nor can be proved by any other evidence which is within reach, nor by any more mode of enquiry [17].

In 1871, the General Committee of The British Association for the Advancement of Science and the British Medical Association adopted principles aimed at the reduction of harm (Refinement), which included: the use of anaesthesia the avoidance of unnecessary repetitions of experiments and that painful research should only be carried out by skilled persons in suitable laboratories under proper regulations [18].

However, it was Russell and Burch who explicitly formulated the Three Rs while working for UFAW, an organisation founded in 1926 by Major Charles Hume to enlist the energies of academics and others with professional expertise in the care of animals, with the aim of using knowledge and the power of science to spread light, rather than heat, on matters of animal welfare. UFAW’s strategy to improve the welfare of animals used in research was to address, first, the husbandry of the animals (during breeding, supply and use); and second, the experimental procedures carried out on the animals.

For the first of these topics, UFAW needed the cooperation of the scientific community. Fortunately, because of its non-confrontational and academic approach, UFAW had already built excellent relationships with those carrying out research on animals. In the words of UFAW’s 1943 Annual Report: “The friendliness of UFAW’s relations with men of science enables us to approach the subject from inside, and we have become aware that many research workers keenly desire to ensure the maximum of consideration for the animals with which they deal”.

As a result, UFAW was able to canvas all 2100 persons in Britain who held a vivisection licence under the 1876 Act, to determine whether there was a demand for a handbook on the care and management of smaller laboratory animals. A positive response was received and in due course (and after disruption from the war, which included some contributors being killed in action) UFAW published the first UFAW handbook on The Care and Management of Laboratory Animals in 1947 [19]. The handbook, now with a ninth edition in preparation, was one of the first of its kind and sought to improve the welfare of animals used in research by improving their care and health, and in doing so, also improve the reliability of results obtained from them.

There remained, however, the need to address the conduct and control of animal experiments. At that time attitudes and practices in research as well as legal controls were very different to today, and some experiments were carried out that caused extreme suffering. Hume, along with several eminent biological scientists (W.R. Wooldridge, A.C. Hardy, E. Hindle, J.R. Baker and F.C. Bartlet), published a letter in the Lancet, 6th August 1949, which described severe and fatal trauma experiments carried out in Canada and America (one on the effect of repeated falls on un-anaesthetised rats, and another that involved the infliction of multiple contusions to dogs’ legs and which resulted in the deaths of 25 out of the 30 animals used in the research). It was against this backdrop that Hume drew attention to the kinds of harms that must be considered in ethical review, and to the importance of animal husbandry. He proposed an increase in the number of Home Office Inspectors (6 at that time), and he and UFAW’s Secretary, foreshadowing later developments in legislation, proposed that the breeding and supply of animals should be centralised to control variation and that experiments should be classified on the basis of their severity [20,21,22]. During the early 1950s, UFAW also supported work on the welfare of laboratory and other animals that included anaesthesia, euthanasia, and the detection of consciousness. These were all important initiatives; however, Hume’s most far-reaching proposal was perhaps his proposal that research was needed on the design and humanitarian aspect of experimental methods and procedures.

Consequently, in July 1954, UFAW appointed Dr William Russell to “undertake research into the history and progress of the introduction of humane methods into biological research with a view to encouraging further such progress.” In a note on the project written in October of the same year, Russell noted the importance of considering stress and distress as well as pain and fear, a point that is incorporated in today’s UK and European legislation, which use the terms “pain, suffering, distress or lasting harm”. He also proposed, and this was absolutely fundamental to the long term impact and lasting benefits of the project, to concentrate on general principles rather than dealing with specific issues, noting that “ it is common scientific experience that the best way to study a new special problem is to find the general one of which it is a special case. Here, clearly the general problem is that of finding the factors promoting accelerating and those obstructing retarding the advance of techniques and experimental biology”.

It soon became clear that Russell needed assistance to carry out the work and UFAW appointed Mr Rex Burch, a microbiologist, to visit and interview research workers regarding their attitudes, the techniques that they had adopted to improve the humaneness of the work they were carrying out, and on the feasibility of replacements to the use of animals [23]. At that time, there was little middle ground between those carrying out research and those who were against it, so this was a difficult task and it appears from Burch’s success that he carried it out with great skill and tact.

According to Bill Russell, the Three Rs evolved as the work progressed over the next two years [24] and, during this period, UFAW records show that there was regular oversight by a UFAW Scientific Sub-Committee chaired by the Nobel Laureate Sir Peter Medawar [25]. By 1955, much of the basis for the Three Rs was already present [26], but the first formal presentation of the Three Rs, albeit concentrating on opportunities for Reduction, was as a presentation at a UFAW Symposium, held in May 1957, on Humane Technique in the Laboratory [27,28].

In 1959, Russell and Burch published The Principles of Humane Experimental Technique [1], in which they clearly laid out the Three Rs for the first time in print. Their use of the words humane and inhumane may appear somewhat confrontational, but they made clear that these terms referred to the effects of different practices on the animals and that they were not intended to reflect on researchers’ behaviour or intentions. The book’s scope was broad, providing firm foundations for the need for change. It explains the ethical concern for the welfare of animals used in research, i.e., the likelihood that some species at least are likely to be conscious. It shows how these affective states are linked to neurological and endocrine system and to changes in physiology caused by stress. It also showed how harms to animals could be graded (as is now done in regulatory systems around the world) and gave practical examples of the use of replacement, reduction and refinement options to minimise harms. Very importantly, Russell and Burch showed that the adoption of humane methods was fundamentally compatible with the objectives of research and conversely that stressed animals would be unlikely to provide good data.

## 3. The Adoption and Spread of the Three Rs

The Principles of Humane Experimental Technique was remarkable, but the Three Rs did not begin to obtain much traction as a concept until the early 1980s. Rex Burch has suggested that delay in uptake may have been because of the newness of the ideas and the negative attitude of a UK committee, set up by the Home Secretary under the Chairmanship of Sir Sydney Littlewood, towards the prospects of finding replacements [23]. Nonetheless, as Stevens describes, there were during the 60s and 70s a number of initiatives to improve the welfare of animals used in research [29] and these combined with increasing concerns about animal welfare in other fields, e.g., Ruth Harrison’s book on farmed animals Animal Machines in 1964, and the rapid development of ethology in the 60s and 70s with associated interest in animal cognition and sentience, may have stimulated renewed interest in the Three Rs. During this period, UFAW continued to work to advance the Three Rs, producing updated editions of the Handbook, supporting work on reduction of experimental variation [30], and running symposia on: assessment of pain (1961); design of small mammal accommodation; and anaesthesia (both in 1963) and the use of animals in toxicology (in 1969). UFAW also recognised the importance of regulation, and following a move in the USA to introduce legal oversight of animal experimentation—the Cooper Bill S.3570—UFAW wrote to congresswoman the Hon. Martha Griffiths pointing to UK scientists’ acceptance of regulation and the benefits to scientists of being regulated:

“Replies to a questionnaire recently issued by UFAW to the most eminent experimental biologists in Britain showed an overwhelming consensus of opinion in favour of the control of experiments as exercised in this country by the Home Office. By ensuring a high standard of moral responsibility in dealing with all species of vertebrates the Home Office also protects scientists from unjust allegations of cruelty” [31].

In the 1980s, the pace of change increased. Interest developed amongst regulators, industry and scientists in finding alternatives to regulatory toxicology tests, while legislative changes occurred in Switzerland and the Federal Republic of Germany requiring consideration of alternatives [29]. In November 1986 The European Community enacted legislation (European Directive 86/609/EEC) which, while it did not explicitly mention the Three Rs, did require member states to implement national legislation that put into practice much of the Three Rs principles. For example, the Directive’s Article 7.4 that all experiments shall be designed to avoid distress and unnecessary pain and suffering to the experimental animals; Article 8 that appropriate anaesthesia and analgesia should be used; and Article 23.1 that the Commission and member states should encourage research into the development and validation of alternative techniques. The Directive also had an Appendix that provided basic guidelines and minimum dimensions for animal housing. Throughout the late 1980s and 1990s searches for alternatives to the use of animals continued with conferences and workshops, the establishment of various institutes and information centres and, notably, the establishment of centres for the validation of alternatives (in Europe, ECVAM—The European Centre for the Validation of Alternative Methods) and in the United States of America ICCVAM—the Interagency Coordinating Committee on the Validation of Alternative Methods) [29].

The Three Rs continued to develop traction in the 2000s. Examples of stake-holder and government initiatives include, the European Consensus Platform for 3R Alternatives to Animal Experimentation ECOPA, which was established in December 2000, and acts as an umbrella organisation for national platforms such as Norway’s National Consensus Platform for the advancement of “the 3 Rs” NORECOPA. In the UK, the first government-funded national centre set up to focus on the Three Rs, The NC3Rs, was set up in 2004, which inspired the Danish 3R-Center in 2013 and there are now others, including in Sweden, Belgium, Italy and Canada and it is likely that this trend will continue.

Legislation controlling the use of animals in research has also adopted the Three Rs. In 2010 the 1986 European Directive was updated and replaced by European Directive 2010/63/EU [32], which came into full effect in 2013. This legislation had a specific aim to provide a solid basis for the application of the Three Rs principles in animal experiments, including new measures to improve the protection and well-being of animals used for experiments such as: encouraging the use of alternative methods, updated minimum housing standards, harm-benefit analysis and ethical review. The legislation specifically mentions the Three Rs in several contexts in both the pre-amble and the body of the legislation and requires the Three Rs to be considered in the evaluation of projects for authorisation. The EU has continued to support a variety of measures to advance the Three Rs in recent years, including networking projects, continued support of ECVAM and through research funding programmes to advance the Three Rs, the latest being Horizon 2020 [33].

In the US, Public Health Service (PHS) policy and USDA Animal Welfare Regulations require institutions to ensure that appropriate consideration is given to alternatives for research that causes more than slight or momentary pain or distress in animals, consistent with sound experimental design, and alternatives are described as being framed within the context of the Three Rs [34] (p. 97). (Bayne et al. (In Prep) [35] provide other examples of their uptake around the World: In India, Institutional Animal Ethics Committees (IAEC) approve protocols, and their principle responsibility is to review and authorise these, with due regard for the Three Rs, as well as inspecting the institution’s animal facility at least twice a year. In Australia, a national standard [36] unifies the different States’ Regulations, and includes consideration of the Three Rs and ethical justification of planned experiments. Other countries such as South Africa, Costa Rica and, most recently, China have codes or legislation that either explicitly or implicitly reference the Three Rs.

The Three Rs Principles have also been actively promoted by the laboratory animal science community. Guillén [37], provides regional examples from laboratory animal science associations based in Europe, Asia, South America, and Australia and New Zealand, pointing out that, although variation has resulted from individual national legislative standards, these organisations have an important role in harmonising standards. At a more global level, the Office International des Epizooties (OIE) provides guidance for member states formulating guidance for the use of live animals in research and regulation that describes the Three Rs and promotes their implementation in ethical review of animal studies [37]. Similarly, the Guiding Principles for Biomedical Research Involving Animals published in 2012 by The Council for International Organization of Medical Sciences and The International Council for Laboratory Animal Science (CIOMS/ICLAS) [38] state:

“The principles of the Three Rs—Replacement, Reduction and Refinement—should be incorporated into the design and conduct of scientific and/or educational activities that involve animals.”

This support from the academic community derives partly from the ethical imperative but also because as Russell and Burch foresaw, more humane methods often facilitate good science, resulting in better, cheaper, or easier outcomes [39] (Chapter 7, pp. 205–225). Examples of such benefits include: the use of replacement in vitro or in silico models which can be much cheaper and quicker than animal models; good experimental design (including power analysis using existing data to determine appropriate sample-size) and better health status, both of which can reduce the numbers of animals needed to obtain data of the required quality and result in savings in animal purchase and care costs; technologies, such as imaging can also reduce the numbers of animals used in studies, the invasiveness of those studies and produce better quality data; while refinement of husbandry and care can reduce stress responses that may confound experimental outcomes [40]. On this last point, even handling methods can have a profound effect on stress responses as has been shown for mice, (which are much less stressed if handled using tubes or cupped hands than if picked up by their tails) [41]. Similarly, for years, in many countries, mice have been housed in grid-bottomed cages which do not easily allow provision of nesting material. However, studies have shown that the temperatures at which mice are housed are outside their thermoneutral zone, and have demonstrated the importance of nesting material, which allows thermoregulation thus avoiding physiological stress [42,43,44,45]. Scientists are increasingly being encouraged to consider the Three Rs not just at the outset of their research, but continuously during it and to improve their reporting of the conduct of their research and of any implications for the Three Rs [46], and by doing so they improve the replicability of their research.

These and other benefits have encouraged the acceptance of the Three Rs amongst the research community. However, although the Three Rs Principles appear simple, they are not always well understood. Application of the Reduction principle, for example, means using the minimum number of animals compatible with obtaining data of sufficient quality, not that fewer animals should always be used. Some Refinements described in project applications turn out, on examination, to be refinements of procedure or experiment rather than improvements in animal welfare. Implementation of the Three Rs may also be less than straightforward as promotion of one R can have an adverse consequence for another R. An example being where an alternative procedure allows fewer animals to be used but causes greater harm to those remaining animals [47]. (In such circumstances, the choice is usually made to give precedence to Refinement over Reduction). Training that incorporates the Three Rs should, therefore, be standard for all new researchers who plan to use animals.

## 4. The Future

Looking to the future, it seems likely that replacements will continue to be found for specific uses of animals but there may be some areas where replacement is difficult or perhaps even impossible. Replacements tend to be specific to the type of study and as new areas of research are developed, there may be new demands for animal use and some of these may have significant welfare impacts. The future, therefore, holds threats to animal welfare, but there are also striking opportunities to advance the Three Rs. Better experimental design and statistical approaches can reduce animal use e.g., [48]. Data and tissue-sharing offer opportunities for more efficient use of information collected from animals and may avoid unnecessary repetition. With respect to refinement, researchers are increasingly implementing training protocols, so that animals cooperate, which reduces the stress of routine procedures, for example blood sampling, and are working on better methods of assessing cumulative impacts of research protocols on welfare, for example through better scoring systems [49] and, possible new markers of stress such as telomere attrition [50] and hippocampal neurogenesis [51,52]. Anaesthesia is now standard [53], but itself can also have a welfare impact [54,55], needs to be carried out well and by trained personnel and there is still work to be done to develop appropriate analgesia regimens. Minimum standards for housing that aim to meet animals’ needs, where necessary with environmental enrichments, are now specified in codes of practice and regulations in many countries and will be even more widely adopted as concerns about the impact on experimental outcomes are addressed [56].

## 5. Conclusions

The Three Rs came about because of: UFAW’s non-confrontational and academic approach to animal welfare issues, Russell and Burch’s vision and decision to concentrate on general rather than specific issues, and through the cooperation of researchers. The Three Rs have provided a clear set of directions for improving the welfare of animals used in research and help to improve scientific outcomes. They have raised the profile of animal welfare amongst biomedical researchers and stimulated research on alternatives, more humane methodologies and better housing and husbandry. The Three Rs provide an ethical framework for research that also provides some reassurance to the public.

Implementation of the Three Rs still varies across the world. Sixty years have passed since their publication but the journey has not ended. Continuing education, investment, and attention to the Three Rs is needed to ensure that use of animals in research is minimised and that the welfare of those animals that are used is maximised.
